# Galectin-3 is related to right ventricular dysfunction in heart failure patients with reduced ejection fraction and may affect exercise capacity

**DOI:** 10.1038/s41598-020-73634-8

**Published:** 2020-10-07

**Authors:** Beata Zaborska, Grażyna Sygitowicz, Krzysztof Smarż, Ewa Pilichowska-Paszkiet, Andrzej Budaj

**Affiliations:** 1 Department of Cardiology, Centre of Postgraduate Medical Education, Grochowski Hospital, Grenadierów 51/59, 04-073 Warsaw, Poland; 2 Department of Clinical Chemistry and Laboratory Diagnostics, Medical University of Warsaw, Warsaw, Poland

**Keywords:** Biomarkers, Cardiology

## Abstract

Galectin-3 is a biomarker of fibrosis, inflammation and oxidative stress, and its role in heart remodelling and exercise intolerance has not been conclusively proven in heart failure (HF) patients with reduced ejection fraction (rEF). We prospectively assessed 67 consecutive patients with symptomatic HF and left ventricular (LV) EF ≤ 35% during optimal medical therapy, with a mean serum galectin-3 concentration of 15.3 ± 6.4 and a median of 13.5 ng/mL. The group with galectin-3 concentrations greater than or equal to the median had significantly worse right ventricular (RV) systolic function parameters (s′, TAPSE), higher pulmonary artery systolic pressure, more advanced tricuspid regurgitation and lower RV-to-pulmonary circulation coupling index, while no significant differences were found in LV parameters. Moreover, this group achieved significantly lower parameters in cardiopulmonary exercise testing. Significant negative correlations were found between galectin-3 concentration and RV parameters and exercise capacity parameters and have persisted after adjustment for glomerular filtration rate, but not all of them have persisted after adjustment for NT-proBNP. Multivariate regression analysis revealed that TAPSE (β coefficient: − 0.605; *p* < 0.001) and heart rate at peak exercise (β coefficient: − 0.98; *p* = 0.009) were independently related to galectin-3 concentration. Elevated galectin-3 concentration in patients with HFrEF might indicate concomitant RV dysfunction and exercise intolerance.

## Introduction

Heart failure (HF) remains one of the most prevalent and complex clinical syndromes with various stages and presentations. Despite improvements in medical therapy, outcomes remain poor, especially in symptomatic patients with severely reduced ejection fraction (rEF).

There is extensive research on biomarkers in HF, and galectin-3 (Gal-3) has recently gained interest. Galectin-3, a soluble β-galactoside-binding lectin, is an important member of the lectin family. It is expressed in various cell types, including fibroblasts, endothelial cells and inflammatory cells^1^. In the failing heart, Gal-3 is released by activated cardiac macrophages and cardiac fibroblasts^1,2^. Galectin-3 is involved in numerous physiological and pathological processes, including inflammation, fibrosis, immunity and oxidative stress, which appears to stimulate pathological remodelling and fibrogenesis, particularly by inducing fibroblast proliferation and collagen deposition^1,3–6^. Therefore, Gal-3 is a “culprit” biomarker in HF and not simply a “bystander” like N-terminal pro B-type natriuretic peptide (NT-pro BNP) or C reactive protein. Although an increased Gal-3 concentration was found to be an independent predictor of mortality and HF hospitalization in chronic HF, the role of Gal-3 in remodelling has not been conclusively demonstrated in patients with heart failure with reduced ejection fraction (HFrEF)^4,7,8^.

Comprehensive echocardiography allows precise heart remodelling and functional assessment. Current guidelines for the diagnosis and treatment of acute and chronic HF emphasize that, along with left ventricular (LV) assessment, assessment of right ventricular (RV) structure and function, including the estimation of pulmonary arterial pressure, is an obligatory element of echocardiographic examination. Recently, in experimental data, an association between Gal-3 and pulmonary artery hypertension was observed^9,10^. This finding was also confirmed in patients with hypoxia-induced pulmonary artery hypertension, those with congenital heart diseases and young patients with obesity. Few small studies have revealed a role of Gal-3 in RV remodelling and dysfunction induced by pulmonary arterial hypertension^10–12^, and there are no published data in patients with HFrEF addressing this issue.

Diminished exercise capacity is a key symptom in HF. Previous studies have demonstrated that RV dysfunction is associated with exercise intolerance in HF patients^13^. In addition, data on the relationship between Gal-3 and exercise capacity (EC) in HF patients are scarce and ambiguous^14–17^. Therefore, the aim of this study was to investigate the relationship of Gal-3 concentration with echocardiographic parameters of LV and RV geometry and function with regard to EC expressed by cardiopulmonary exercise testing (CPX) parameters in patients with chronic HFrEF.

## Results

### Patient characteristics

The study group consisted of 67 patients (81% males, mean age 67 ± 9 years), and seventy-five percent of patients had ischaemic cardiomyopathy. All patients were symptomatic, in NYHA class II or III, despite optimal medical therapy. The mean Gal-3 concentration was 15.3 ± 6.4, with a median of 13.5 ng/mL. Table 1 shows a comparison of demographic and clinical characteristics of the study group in relation to Gal-3 concentration. No significant differences were found regarding clinical status, comorbidities, risk factors, ECG, haemoglobin level, platelets count, glomerular filtration rate (GFR) and creatinine concentration. Significant differences between groups were found regarding total bilirubin and alanine aminotransferase (ALT). Also, INR and Model for End Stage Liver Disease incorporating Sodium (MELD-Na) were higher in the group with higher Gal-3. Other liver function parameters and indexes of liver cirrhosis and fibrosis expressed by Model for End Stage Liver Disease eXcluding INR (MELD-XI) and Fibrosis-4 (FIB-4) respectively, were comparable between groups. NT-pro BNP level tended to be higher in patients with Gal-3 concentrations at or above the median, although the difference did not reach statistical significance.

**Table 1:** Demographic and clinical characteristics of the study group in relation to galectin-3 concentration.

	All patients n = 67	Gal-3 < med n = 33	Gal-3 ≥ med n = 34	*p*-value
Age	67 ± 9	68.6 ± 7.4	64.8 ± 10.0	0.10
Sex (male/female)	54/13	29/4	25/9	0.22
Systolic blood pressure (mmHg)	120 ± 17	121.4 ± 15.1	118.1 ± 18.5	0.43
Diastolic blood pressure (mmHg)	74 ± 8	74.5 ± 8.3	73.1 ± 8.5	0.47
Body mass index (kg/m^2^)	28 ± 4	27.2 ± 3.6	28.5 ± 4.7	0.22
**NYHA class, n (%)**				0.18
II	27 (40)	17 (52)	10 (29)	
III	40 (60)	16 (48)	24 (71)	
**Aetiology of heart failure, n (%)**				0.83
Ischaemic	50 (75)	25 (76)	25 (74)	
Non-ischaemic	17 (25)	8 (24)	9 (26)	
Active smokers, n (%)	12 (18)	6 (18)	6 (18)	0.95
Former smokers, n (%)	20 (30)	11 (33)	9 (26)	0.54
**Past medical history, n (%)**				
Myocardial infarction	44 (66)	21 (64)	23 (68)	0.73
Hypertension	49 (73)	22 (67)	27 (79)	0.24
Diabetes mellitus	26 (39)	10 (30)	16 (47)	0.16
Dyslipidaemia	27 (40)	15 (45)	12 (35)	0.40
**ECG rhythm**				0.13
Sinus rhythm	59 (87)	31 (94)	28 (82)	
Atrial fibrillation	8 (11)	2 (6)	6 (18)	
ECG QRS duration (ms)	157 ± 30	162.4 ± 28.6	152.4 ± 30.4	0.10
**ECG QRS morphology**				0.60
LBBB	33 (49)	17 (52)	16 (47)	
RBBB	3 (4)	1 (3)	2 (6)	
Haemoglobin (g/dl)	14 ± 2	13.5 ± 1.0	13.8 ± 2.0	0.52
Platelets (10^9^/L)	226 ± 84	216 ± 55	236 ± 105	0.81
Creatinine (mg/dL)	1.27 ± 0.3	1.22 ± 0.24	1.32 ± 0.35	0.18
GFR (ml/min)	63.6 ± 24.1	62.9 ± 22.5	64.4 ± 25.8	0.88
Sodium (mmol/L)	139.8 ± 3.5	140,3 ± 2.7	139.3 ± 4.1	0.24
NT-pro BNP (pg/ml)* (p25-p75)	1605 (731- 3334)	1313 (634–2216)	1957 (881–3588)	0.13
**Liver function**				
Total bilirubin (mg/dL)	1.1 ± 0.3	1.0 ± 0.3	1.2 ± 0.4	0.023
AST (U/L)	30 ± 17	28 ± 13	32 ± 21	0.14
ALT (U/L)	35 ± 22	29 ± 17	41 ± 26	0.007
INR	1.2 ± 0.4	1.1 ± 0.2	1.3 ± 0.5	0.021
**Scores**				
MELD-Na	11.5 ± 4.7	9.9 ± 3.2	13.0 ± 5.3	0.005
MELD-XI	12.2 ± 3.6	11.5 ± 2.7	12.9 ± 4.2	0.11
**FIB-4, n (%)**				0.18
< 1.45	31(46)	13 (39)	18 (53)	
1.45–3.25	32 (48)	17 (52)	15 (44)	
> 3.25	4 (6)	3 (9)	1 (3)	
**Medications (%)**				
Diuretics	67 (100)	33 (100)	34 (100)	
ACE inhibitors	55 (82)	28 (85)	27 (79)	0.56
ARBs	9 (13)	4 (12)	5 (15)	1.0
Beta-blockers	66 (99)	33 (100)	33 (97)	1.0
Antiarrhythmics	21 (31)	10 (30)	11 (32)	0.86
Aldosterone antagonists	46 (69)	24 (73)	22 (65)	0.48
Statins	54 (81)	29 (88)	25 (74)	0.22
Digoxin	9 (13)	2 (6)	7 (21)	0.15
Oral anticoagulants	20 (30)	6 (18)	14 (41)	0.04

Values are expressed as the mean ± SD and (%) and range or number.

*Values are expressed as the median with interquartile range.

Gal-3, galectin-3; NYHA, New York Heart Association; LBBB , left bundle brunch block; RBBB, right bundle brunch block; GFR, glomerular filtration rate; NT-pro BNP, N-terminal pro B-type natriuretic peptide; AST, aspartate aminotransferase: ALT, alanine aminotransferase; INR, international normalized ratio; MELD-Na, Model for End Stage Liver Disease incorporating Sodium; MELD-XI, Model for End Stage Liver Disease eXcluding INR; FIB-4, Fibrosis—4; ACE inhibitors, Angiotensine converting enzyme inhibitors; ARBs, angiotensin receptor blockers.

Patients with higher Gal-3 were more frequently treated with oral anticoagulants.

### Relationship between Gal-3 and LV geometry and function

All patients presented severe LV systolic dysfunction with a mean LV EF of 26 ± 6% and a mean LV global longitudinal strain (GLS) of − 7 ± 3%. Diastolic dysfunction was present in 59 (88%) patients. Echocardiographic parameters are shown in Table 2. No significant differences were found in LV diameter, systolic and diastolic function, or degree of mitral regurgitation between the group with Gal-3 concentration less than the median and the group with Gal-3 concentration greater than or equal to the median. Moreover, none of the assessed LV echocardiographic parameters correlated with Gal-3 concentration (Table 3).

**Table 2:** Comparison of echocardiographic measurements in patients with different levels of galectin-3.

Variables	All patients n = 67	Galectin-3 < median n = 33	Galectin-3 ≥ median n = 34	*p*-value
RV inflow diameter (cm)	4.0 ± 0.7	3.9 ± 0.6	4.1 ± 0.8	0.18
RV outflow diameter (cm)	3.2 ± 0.6	3.0 ± 0.4	3.3 ± 0.6	0.010
RV sʹ (cm/s)	10 ± 3	10.7 ± 2.6	9.5 ± 2.6	0.042
RV eʹ (cm/s)	8 ± 4	8.1 ± 3.0	8.3 ± 4.0	0.82
TAPSE (mm)	19.1 ± 4.6	20.9 ± 4.7	17.4 ± 3.9	0.0016
RV fraction area change (%)	38.6 ± 13.0	40.4 ± 13.4	36.8 ± 12.6	0.26
RV 2D strain (%)	− 13 ± 6	− 13.8 ± 4.4	− 12.6 ± 4.1	0.07
RV free wall 2D strain (%)	− 16 ± 8	− 17.2 ± 6.3	− 16.6 ± 6.1	0.83
TAPSE/PASP	0.69 ± 0.36	0.85 ± 0.34	0.54 ± 0.3	0.0004
LV ejection fraction (%)	26 ± 7	26.5 ± 7.4	25.2 ± 6.6	0.24
LV end diastolic volume (ml)	206 ± 63	209 ± 64	206 ± 60	0.91
LV end systolic volume (ml)	156 ± 58	157 ± 59	156 ± 56	0.96
dP/dt (mmHg/s)	548 ± 140	541 ± 143	554 ± 139	0.67
LV sʹ (cm/s)	4.2 ± 1.2	4.2 ± 1.3	4.1 ± 1.1	0.85
LV GLS (%)	− 7 ± 3	− 6.8 ± 2.7	− 7.4 ± 2.6	0.29
**Mitral regurgitation, n (%)**				0.87
None/Mild	35 (52)	20 (61)	17 (50)	
Moderate	21 (31)	9 (27)	12 (35)	
Severe	9 (13)	4 (12)	5 (15)	
**Tricuspid regurgitation, n (%)**				0.01
None/Mild		29 (88)	19 (56)	
Moderate		3 (9)	13 (38)	
Severe		1(3)	2 (6)	
LV E/eʹ	16.8 ± 7.4	15.5 ± 7.0	18.1 ± 7.7	0.22
PASP (mmHg)	35 ± 18	29.1 ± 14.9	40.1 ± 18.8	0.007
RAP (mmHg)	5.9 ± 4.5	4.6 ± 3.3	7.1 ± 5.2	0.048
LAVi (ml/m2)	45.4 ± 15.8	43.2 ± 14.3	47.6 ± 17.1	0.19
**LV diastolic dysfunction, n (%)**				0.49
Grade 1	28 (42)	17 (55)	11 (39)	
Grade 2	13 (19)	6 (19)	7 (25)	
Grade 3	18 (27)	8 (26)	10 (36)	

Values are expressed as the mean ± SD and (%) and range or number.

RV, right ventricular; sʹ, systolic myocardial velocity; eʹ, early diastolic myocardial velocity; TAPSE, tricuspid annular plane systolic excursion; PASP, systolic pulmonary artery pressure; RAP, right atrial pressure; LV, left ventricular; GLS, global longitudinal strain; E/eʹ, ratio of early diastolic transmitral velocity to peak early diastolic myocardial velocity; LAVi, left atrial volume index.

LV diastolic dysfunction assessment was performed for patients in sinus rhythm.

**Table 3:** Correlations between galectin 3 and echocardiografic parameters of left and right ventricular function and parameters of exercise capacity derived from CPX.

Parametres	Correlation		Correlation adjusted for NTproBNP		Correlation adjusted for GFR	
	rho	*p*	rho	*p*	rho	*p*
**Left ventricular function**						
LV ejection fraction	− 0.06	0.64	0.01	0.95	− 0.10	0.42
LV sʹ	0.03	0.78	0.15	0.23	0.04	0.78
dP/dt	0.09	0.46	0.17	0.17	0.10	0.43
LV end diastolic volume	− 0.06	0.57	− 0.14	0.25	− 0.02	0.88
LV end sysytolic volume	− 0.06	0.66	− 0.14	0.26	− 0.01	0.92
LV strain	− 0.18	0.18	**− 0.27**	**0.042**	− 0.14	0.30
E/e’	0.16	0.21	0.004	0.97	0.14	0.27
Left atrial volume index	0.19	0.13	0.08	0.55	0.18	0.14
**Right ventricular function**						
RV fraction area change	− 0.08	0.50	− 0.6	0.66	− 0.12	0.35
TAPSE	**− 0.33**	**0.006**	**− 0.29**	**0.018**	**− 0.35**	**0.004**
RV sʹ	**− 0.25**	**0.04**	− 0.20	0.12	**− 0.25**	**0.044**
RV eʹ	− 0.02	0.85	− 0.07	0.60	− 0.01	0.92
PASP	**0.32**	**0.008**	0.21	0.08	**0.33**	**0.008**
RV 2D strain	0.15	0.26	0.02	0.89	0.21	0.12
RV free wall 2D strain	0.04	0.77	− 0.05	0.70	0.04	0.76
TAPSE/PASP	**− 0.41**	**0.00064**	**− 0.32**	**0.009**	**− 0.42**	**0.000**
**Exercise capacity**						
Exercise time	**− 0.31**	**0.011**	**− 0.26**	**0.034**	**− 0.32**	**0.010**
HR at peak exercise	**− 0.27**	**0.027**	− 0.21	0.097	**− 0.25**	**0.047**
HR peak/max predicted	**− 0.29**	**0.015**	− 0.24	0.058	**− 0.29**	**0.017**
Systolic blood pressure peak	− 0.19	0.12	− 0.06	0.61	− 0.20	0.11
Diastolic blood pressure peak	− 0.068	0.58	0.00	0.97	− 0.05	0.72
RER	− 0.18	0.14	**− 0.25**	**0.04**	− 0.18	0.15
Peak VO_2_/kg	**− 0.25**	**0.04**	− 0.17	0.17	**− 0.27**	**0.03**
AT/kg	− 0.19	0.12	− 0.09	0.50	− 0.23	0.07
VE/VCO_2_ slope	0.20	0.098	0.01	0.48	0.20	0.11

LV, left ventricular; RV, right ventricular; sʹ, systolic myocardial velocity; eʹ, early diastolic myocardial velocity; E/eʹ, ratio of early diastolic transmitral velocity to peak early diastolic myocardial velocity; TAPSE, tricuspid annular plane systolic excursion; PASP, systolic pulmonary artery pressure; HR, heart rate; RER, respiratory exchange ratio at peak exercise; peak VO_2_/kg oxygen uptake at peak exercise; AT/kg oxygen uptake at anaerobic threshold.

Bold values are statistically significant.

### Relationship between Gal-3 and RV geometry and function

A wide range of RV function was observed in the study group, from patients with parameters within normal limits to those with severely depressed parameters. The group with Gal-3 concentrations greater than or equal to the median had significantly worse RV long-axis function parameters, more advanced tricuspid regurgitation and higher right heart pressures expressed as pulmonary artery systolic pressure (PASP) and right atrial pressure (RAP). Additionally, the tricuspid annular plane systolic excursion (TAPSE)/PASP ratio, a non-invasive index of RV-to-pulmonary circulation coupling, was significantly lower; these data are presented in Table 2. Significant, moderate correlations were found between Gal-3 concentration and the abovementioned RV parameters (Table 3). All found correlations, regarding clinical and statistical significance, have persisted after adjustment for GFR. After adjustment for NT-proBNP, correlations with RV function expressed as TAPSE and TAPSE/PASP have persisted, but correlations with RV s’ and PASP have not. Backward multivariate linear regression analysis revealed that RV systolic function assessed as TAPSE was independently negatively related to Gal-3 concentration (β coefficient: − 0.605; 95% CI 0.90 to − 0.31; *p* < 0.001) (Table 4).

**Table 4:** Backward multivariate linear regression analysis of the Gal-3 relationship with clinical characteristics, RV echocardiographic parameters and EC parameters.

Variable	Coeff	95%CI	P value
TAPSE/PASP			0.96*
NT-proBNP/1000			0.96*
VE/VCO_2_ slope			0.78*
Total bilirubin			0.61*
Glomerular filtration rate			0.59*
RV outflow diameter			0.54*
PASP			0.40*
VO_2_ pulse			0.39*
Alanine aminotransferase			0.38*
pVO_2_			0.38*
FIB-4			0.15*
RV s′			0.14*
MELD-XI			0.11*
TAPSE	− 0.605	− 0.90 to-0.31	< 0.001
HR at peak exercise	− 0.098	− 0.17 to-0.26	0.009

*Variables with *p*-value > 0.1 were considered nonsignificant.

TAPSE, tricuspid annular plane systolic excursion; PASP, systolic pulmonary artery pressure; NT-pro BNP, N-terminal pro B-type natriuretic peptide; VE/VCO_2,_ minute ventilation-carbon versus carbon dioxide production; Peak VO_2,_ oxygen uptake at peak exercise_;_ FIB-4, Fibrosis-4_;_ RV, right ventricular; sʹ, systolic myocardial velocity; MELD-XI, Model for End Stage Liver Disease eXcluding INR;HR, heart rate.

### Relationship between Gal-3 and EC

We compared CPX parameters in relation to the Gal-3 concentration (Table 5). We found that the oxygen uptake at peak exercise (peak VO_2)_ and at the anaerobic threshold were significantly lower in patients with a Gal-3 concentration at or above the median. Exercise duration was shorter, and peak systolic blood pressure was lower in this group than in the group with Gal-3 concentrations lower than the median. Additionally, the percentage of predicted maximal heart rate (HR) achieved at peak exercise was lower in patients with higher Gal-3 concentrations. The investigated groups did not differ either in respiratory exchange ratio at peak exercise or in ventilatory response to exercise assessed in minute ventilation-carbon versus carbon dioxide production (VE/VCO_2_) slope. Furthermore, no pulmonary limitations of exercise were noted in any patients. Breath reserve at peak exercise was preserved in both groups. Significant but weak negative correlations were found between Gal-3 concentration and exercise duration, HR at peak exercise, percentage of predicted maximal HR, and peak VO_2_ (Table 3). All found correlations, regarding clinical and statistical significance, have persisted after adjustment for GFR. Partial correlations with EC parameters adjusted for NT-proBNP revealed significance only for exercise duration and respiratory exchange ratio. Backward multivariate linear regression analysis revealed that Gal-3 concentration was independently negatively associated with HR at peak exercise (β coefficient: − 0.98; 95% CI − 0.17 to − 0.26; *p* = 0.009) (Table 4).

**Table 5:** Comparison of cardiopulmonary exercise testing parameters in relation to galectin-3 concentration.

Variables	All patients n = 67	Galectin-3		*p*-value
		< median n = 33	≥ median n = 34	
Exercise time (min)	5.8 ± 3.7	7.0 ± 4.1	4.6 ± 2.9	0.014
Heart rate at rest (beats/min)	77 ± 14	78 ± 16	76 ± 12	0.91
Heart rate at peak exercise (beats/min)	106 ± 19	110 ± 19	103 ± 19	0.16
Heart rate at peak exercise/maximal predicted (× 100) (%)	77 ± 15	81 ± 14	74 ± 15	0.038
Systolic blood pressure at peak exercise (mmHg)	136 ± 24	142 ± 20	130 ± 25	0.041
Diastolic blood pressure at peak exercise (mmHg)	72 ± 9	72 ± 11	72 ± 8	0.92
Respiratory exchange ratio at peak exercise	1.05 ± 0.10	1.07 ± 0.09	1.03 ± 0.11	0.11
Oxygen uptake at peak exercise (ml/kg/min)	13.9 ± 5.0	15.3 ± 5.7	12.5 ± 3.9	0.020
Oxygen uptake at anaerobic threshold (ml/kg/min)	12.4 ± 3.9	13.5 ± 4.1	11.4 ± 3.4	0.029
VE/VCO_2_ slope	31.1 ± 7.1	30.3 ± 7.3	32 ± 6.8	0.35
VO_2_ pulse	18.6 ± 4.9	17.6 ± 4.4	19.6 ± 5.3	0.022
Breath reserve at peak exercise (%)	43 ± 19	39 ± 21	46 ± 15	0.11

Values are expressed as the mean ± SD and (%) and range or number.

## Discussion

The major finding of this study is that in symptomatic patients with chronic HFrEF, elevated Gal-3 concentration is related to worsened RV function but not LV function. To our knowledge, this is the first study on this group of patients demonstrating an impairment in RV long-axis function, higher PASP and increased RAP and therefore a lower index of RV-to-pulmonary circulation coupling (TAPSE/ PASP ratio), which is one of the most important non-invasive indexes in terms of RV failure assessment. In the group with higher Gal-3 concentrations, we observed a decline in EC expressed as lower oxygen uptake at peak exercise and at the anaerobic threshold in CPX. Negative associations of Gal-3 with TAPSE and HR at peak exercise were found in backward multivariate linear regression analysis.

Although there are data on the association between galectin-3 and renal function, in our study all found correlations persist after adjustment for GFR^18^. A possible explanation is that all patients from our study group were in chronic but stable condition, during carefully controlled optimal medical therapy. All of them were treated with diuretics.

RV function has proven to be a prognostic factor in HF with preserved and reduced EF^19,20^. In HFrEF, the development of RV HF is observed due to pressure overload resulting from an increase in pulmonary hypertension, which leads to a gradual increase in RV afterload and volume overload, mainly due to tricuspid regurgitation. Right ventricular dilation and a decline in longitudinal function are the main steps in RV failure development^21^. All the elements of the abovementioned pathology were observed in our study group with higher Gal-3 concentrations. Several potential mechanisms underlying the relationship between Gal-3 and RV function have been postulated and vigorously investigated. Right ventricle remodelling and fibrosis may lead to RV dysfunction. Crnkovic et al.^22^ assessed the expression of fibrotic markers in the right ventricle of patients with pulmonary hypertension and in experimental animal models. Established fibrosis was found to be characterized by marked expression of Gal-3 and enhanced numbers of proliferating RV fibroblasts^22^. In an animal model, Hao et al.^9^ demonstrated that Gal-3 inhibition ameliorated hypoxia-induced pulmonary artery hypertension and reduced the inflammatory response, and they showed the genetic and molecular pathways involved. In congenital heart disease patients and mouse models, Shen et al.^10^ identified facilitating roles of Gal-3 in pulmonary artery remodelling and the progression of pulmonary artery hypertension. Moreover, in a study on patients with pulmonary artery hypertension and in an animal model, He et al.^11^ found that Gal-3-mediated pulmonary artery hypertension induced RV remodelling by interacting with NADPH oxidase 4 and NADPH oxidase 4-derived oxidative stress. Although patients with primary pulmonary hypertension were not represented in our study group, higher systolic pulmonary artery pressure and higher right atrial pressure were found among patients with Gal-3 concentration at or above the median. Pulmonary hypertension is a powerful predictor of mortality in patients with symptomatic HF^23^, and it also leads to RV dysfunction because the right ventricle tolerates volume better than pressure overload due to RV anatomy. In our study, significantly lower TAPSE/PASP ratios were found in patients with Gal-3 concentrations greater than or equal to the median, suggesting uncoupling of the right ventricle-pulmonary artery. The TAPSE/PASP ratio was significantly correlated with the Gal-3 concentration. A similar correlation was found by Gopal et al.^12^ in young obese patients with metabolic heart disease.

In our study some found correlations between Gal-3 and RV function parameters have not persisted after adjustment for NT-proBNP. Natriuretic peptides and NT-proBNP among them play a crucial role in the diagnosis of HF, especially acutely decompensated. Nevertheless, there are some difficult points in their interpretation. RV dysfunction and pulmonary hypertension are listed as the clinical conditions when NT-proBNP level might be higher than expected. On the other hand, lower NT-proBNP level than expected is associated with end-stage cardiomyopathy^24^.

Advanced heart failure is a complex clinical syndrome, and a condition of other target organs, like liver, must be taken into account, especially in patients with coexisting RV dysfunction. Therefore, we have included liver function parameters into the backward multivariate linear regression analysis model: total bilirubin, ALT, FIB-4 and MELD-XI. We have chosen MELD-XI score because it is relevant concerning oral anticoagulant therapy and its prognostic utility in patients with end-stage heart failure was shown^25^ . In our group none of the liver function parameters or scores was significant.

Additionally, the role of the RV in determining EC has gained much interest. In our previous study on symptomatic patients with chronic HFrEF, we demonstrated that the presence of RV systolic dysfunction was independently related to reduced EC^13^.

In the current study, we found a weak but significant negative correlation between Gal-3 concentration and HR at peak exercise, percentage of predicted maximal HR, VO_2_ at peak exercise, and exercise time. In multivariate regression analysis, HR at peak exercise was independently negatively related to Gal-3 concentration. HR response during exercise is an important factor determining EC, especially in patients with HFrEF and severe LV contractile dysfunction^26,27^. Patients with higher Gal-3 concentrations may have worse HR responses during exercise than those with lower Gal-3 concentrations and therefore worse EC.

Although there are some data that ventilatory efficiency relates to elevated pulmonary pressure and correlates with RV dysfunction, in our study, there were no differences in VE/VCO_2_ slope between groups ^28–30^. It could be due to described stronger correlations between ventilatory efficiency and RV function during exercise which were not analyzed in our study ^28,30^.

NT-pro BNP correlates strongly with exercise tolerance and can predict low EC measured by CPX in patients with impaired LV function ^31,32^ . It is a likely explanation why most of the weak correlations between Gal-3 and exercise capacity parameters have not persisted after adjustment for NT-proBNP.

Exercise intolerance in patients with chronic HF is a strong predictor of survival and HF progression. The relations between inflammatory biomarkers and exercise capacity in HF patients are ambiguous. In a group of 895 HF patients included in the HF-ACTION Study, Felker et al.^14^ found a significant but weak negative correlation between Gal-3 levels and oxygen uptake at peak exercise (r = − 0.25, *p* < 0.001). In contrast, Atabakhshian et al.^16^ reported no significant correlation between Gal-3 levels and functional capacity in 76 patients with compensated HF in NYHA classes I–IV with LV EF < 45% (*p* = 0.420). In this study, functional capacity was assessed as patient ability to perform activities according to NYHA classes^16^. Fernandes-Silva et al.^17^ investigated EC and the response to exercise training programmes according to inflammatory biomarker plasma concentrations in 44 HF patients with LV EF < 40%. The authors found that peak VO_2_ was significantly improved only in patients with low levels of inflammatory biomarkers. Similarly, cardiac rehabilitation improved peak VO_2_ compared with the control condition in patients with a Gal-3 concentration lower than the median (2.4 ± 0.8 vs. 0.3 ± 0.9 ml/kg/min, *p* = 0.032) but not among those with a Gal-3 concentration higher than the median (0.3 ± 0.7 vs. − 0.7 ± 1.0 ml/kg/min, *p* = 0.41, *p* = 0.053)^17^. In another study, Billebeau et al.^15^, in a group of 107 chronic HF patients with LVEF ≤ 45%, showed that improvement of EC after cardiac rehabilitation correlated with a reduction in Gal-3 and other cardiac biomarkers, suggesting an overall improvement of the neuro-hormonal profile. The authors suggested that cardiac rehabilitation could be a therapeutic intervention accompanied by a decrease in Gal-3 concentration^15^.

In our study, Gal-3 concentration did not correlate with the echocardiographic parameters of LV geometry and function. Several processes involved in HF, such as fibrogenesis, myofibroblast proliferation and inflammation, are linked with Gal-3. Gopal et al.^18^ showed that Gal-3 concentration was elevated in patients with both stable and acutely decompensated HFrEF compared to controls. However, there is no consensus in the literature on Gal-3 relevance in LV remodelling and prognostic value in patients with HF. Lisowska et al.^33^ demonstrated no correlation between the LV EF value and Gal-3 concentration in patients with myocardial infarction and stable coronary artery disease. In the study consisting of 240 HFrEF patients with predominant ischaemic aetiology, Lok et al.^34^ found a significant correlation between Gal-3 levels and changes in LV end diastolic volume, but no correlation between Gal-3 levels and LV end diastolic volume value. Elevation of Gal-3 levels and LV remodelling were more prominent in patients with modest LV enlargement than in patients with larger ventricles at baseline, who developed reverse remodelling more frequently. This observation suggests that Gal-3, a marker of inflammation and fibrosis, is especially elevated in HF patients with LV remodelling compared to patients without LV remodelling. Moreover, increased Gal-3 levels were found to be more pronounced in acute HF than in chronic HF, suggesting a moderate inflammatory response in chronic HF compared to that in acute HF^35^. Other studies demonstrated that higher concentrations of Gal-3 were correlated with an increased risk for new HF in healthy people and LV remodelling in acute MI patients with preserved LVEF, suggesting that Gal-3 could play a more important role in early fibrosis at the early stage of HF^8,36^. In contrast, our study group consisted of patients with chronic HF with severe systolic dysfunction and rather enlarged LV volumes. Our findings are in accordance with the observation that Gal-3 is not associated with outcomes in older patients with advanced chronic HF of ischaemic aetiology and therefore may have limited prognostic value in such a group of patients^37^.

The question of values of Gal-3 concentration, normal and abnormal, remains open. It mainly depends on the tested population. In our study, the mean Gal-3 concentration was 15.3 ± 6.4, with a median of 13.5 ng/mL (interquartile range 11.1–18.1). It is in concordance with HF-Action Study, in which a cohort of 895 subjects with chronic HF with systolic dysfunction, EF < 35% were analyzed. The median Gal-3 concentration in this cohort study was 14 ng/mL (interquartile range 11.0–18.6)^14^. The data on values of Gal-3 in healthy subjects are relatively scarce, mainly due to difficulty of gathering truly healthy population, carefully characterizing and representing various age ranges. Results are also sometimes contradictory, with regard to age and sex. Agnello L et al. recruited 706 blood donors and found that median Gal-3 concentration was 14.3 (Interquartile range 11.9–16.7) ng/mL with 97.5th percentile URL 26.1 ng/mL and it was related to age^38^. Mueller T et al. also examined blood donors concluded that 97.5th percentile URL for Gal-3 was 16 ng/mL in males and 17 ng/mL in females^39^ .

## Limitations

Certain limitations of this study should be considered. First, this is a single centre study with a relatively small number of patients. Although our study group was rather homogeneous, as we enrolled candidates for cardiac resynchronization therapy implantation, we must appreciate the complexity of HFrEF pathophysiology. Second, only a single Gal-3 concentration measurement was performed per patient; however, the timing was carefully selected within 24 h with echocardiographic examination and CPX.

## Conclusions

In symptomatic patients with chronic HFrEF, Gal-3 concentration was related to worsened RV, but not LV, function and was accompanied by a lower index of RV-to-pulmonary circulation coupling. Further, Gal-3 concentration negatively correlated with EC, however most of the correlations have not persisted after adjustment for NT-proBNP. Multivariate regression analysis revealed that Gal-3 concentration was negatively related to TAPSE and HR at peak exercise.

### Clinical implication

Elevated Gal-3 concentrations in patients with HFrEF might indicate concomitant RV dysfunction and exercise intolerance. The clinical relevance of this finding needs to be confirmed in further studies.

## Methods

### Study population and design

Consecutive patients with symptomatic HF in New York Heart Association (NYHA) classes II-III and severe LV systolic dysfunction (LV EF ≤ 35%) referred to our Cardiology Department between January 2013 and December 2015 for consideration of cardiac resynchronization therapy were evaluated with echocardiography and CPX within 24-h intervals as we previously described and published^13^. Routine laboratory tests, including measurements of serum NT-proBNP concentrations, were performed immediately, and the remaining serum was frozen for further biochemical analyses. To assess liver function we calculated indexes of cirrhosis and fibrosis using well-defined formulas of the following scores:

$$\begin{aligned}{\text{MELD-XI}} &= {5}.{11} \times \left( {{\ln}\;{\text{of}}\;{\text{total}}\;{\text{bilirubin}}\;{\text{in}}\;{\text{mg/mL}}} \right) + {11}.{76} \times {\ln}\;{\text{of}}\;{\text{creatinine}}\;{\text{in}}\;{\text{mg/mL}}) + {9}.{44}; \\ {\text{MELD-Na}} &= {\text{MELDscore}}{-}{\text{serum}}\;{\text{Na}}{-}\left( {.0{\text{25 X MELDscore}} \times \, \left( {{14}0 - {\text{Na}}} \right)} \right) + {14}0 \end{aligned}$$

where MELD score = 11.2 ln of INR + 3.78 ln of total bilirubin + 9.57 ln of creatinine + 6.43.

$${\text{FIB-4}} = \left({\text{age}}[\text{years}] \times \text{aspartate}~\text{aminotransferase}[\text{U}/\text{L}]\right)/\left({\text{platelets}}[10^{9}/\text{L}] \times \text{alanine}~\text{aminotransferase}^{1/2}[\text{U}/\text{L}]\right)$$^40–42^.

All patients remained on optimal medical therapy with no further options for myocardial revascularization or other aetiological HF treatment^13^. The exclusion criteria were haemodynamic instability, severe organic valvular disease, chronic lung diseases, inability to perform an exercise treadmill test^13^. All 67 patients enrolled in this observational study were prospectively assessed. A detailed description of the methods and results was published earlier^13^. The Gal-3 concentrations of the previously frozen serum samples were measured, and analyses of the association of Gal-3 concentration with echocardiographic parameters, namely, LV and RV geometry and function and EC, were performed.

This study was performed in accordance with the requirements of the Declaration of Helsinki. The study and all its protocols were approved by the Bioethical Committee of the Centre of Postgraduate Medical Education. All the participants provided written informed consent prior to the inclusion to the study.

### Echocardiographic assessment

Echocardiographic examinations were performed with a Vivid E9 ultrasound machine (GE Healthcare, Horten, Norway). All measurements were performed by an experienced cardiologist according to the recommendations of the American Society of Echocardiography, the European Association of Cardiovascular Imaging and the guidelines for echocardiographic assessment of the right heart^43,44^. Detailed assessment of LV geometry, systolic and diastolic function derived from 2D echocardiography, Doppler examination, tissue Doppler imaging and 2D speckle tracking echocardiography was performed. LV EF was calculated according to the modified Simpson’s rule. Other assessed parameters included dP/dt, LV diastolic dysfunction grade, E/eʹ ratio, left atrial maximum volume index (LAVi), mitral regurgitation grade, peak systolic myocardial velocity (LV s′), and LV GLS.

RV geometry and function assessment was based on M-mode, 2D echocardiography, Doppler examination, tissue Doppler imaging and 2D strain assessment. Measurements including fractional area change (FAC) in the RV, TAPSE, peak systolic myocardial velocity (sʹ) and peak early (e′) and late diastolic velocities at the RV, RV systolic longitudinal 2D strain, RV free wall 2D strain and assessment of RV wall motion abnormalities were carried out. Systolic pulmonary artery pressure (PASP) was estimated. The TAPSE/PASP ratio was used as a non-invasive index of RV-to-pulmonary circulation coupling.

### Exercise capacity

A symptom-limited treadmill CPX with a Schiller Cardiovit CS-200 (Schiller, Baar, Switzerland) and an Ergo Spiro adapter (Ganshorn, Niederlauer, Germany) was performed at the same time of the day (between 11 am and 1 pm). The Naughton or modified Bruce protocol was used, depending on the patient’s functional status. Oxygen uptake at peak exercise in mL/kg/min was used as EC parameter. Other analysed exercise parameters were oxygen uptake at the anaerobic threshold, minute ventilation, ventilatory reserve at peak exercise, VE/VCO_2_ slope, exercise duration, HR at peak exercise, percent of maximum predicted HR at peak exercise, and blood pressure at peak exercise. VE/VCO2 slope (VE plotted versus VCO2 slope) was calculated by linear regression from rest to peak exercise. All CPX was performed according to the American Thoracic Society/American College of Chest Physicians Guidelines^45^.

### Measurements of galectin-3 concentration

Serum Gal-3 concentration was measured using reagents and an instrument in the VIDAS family (bioMerieux SA, Marcy-I`Etoile, France)^46,47^. The assay principle combines a one-step immunoassay sandwich method with final fluorescence detection (ELFA—enzyme-linked fluorescence assay). Assay calibration was performed according to the manufacturer’s recommendations, and values were normalized to the standard curve^47^. This assay has a measurement range of 3.3 to 100 ng/mL, high repeatability (CV approximately 1%) and reproducibility (CV approximately 5%). The intra-assay and inter-assay variances for Gal-3 were 1.25% and 5.5%, respectively^47^.

### Statistical analysis

Categorical variables are presented as numbers and percentages. Continuous variables are presented as the mean ± standard deviation (SD) or as the median when appropriate. Continuous variables were compared using Student’s t-test or the Mann–Whitney U test for non-parametric data. A *p*-value < 0.05 was considered statistically significant. Spearman's correlation coefficients were calculated to assess the relationship between continuous variables. Partial correlations between galectin-3 and examined variables after adjustment for GFR and NT-proBNP were assessed. Backward multivariate linear regression analysis was performed to explore the relationships between Gal-3 concentration and clinical characteristics, RV echocardiographic parameters and EC parameters. The following parameters were included in the model: plasma NT-proBNP, GFR, ALT, total bilirubin, MELD-XI, FIB-4, RV outflow diameter, TAPSE, RV s′, PASP, TAPSE/PASP, peak VO_2,_ HR at peak exercise, VO_2_ pulse and VE/VCO_2_ slope. The analyses were carried out using STATA 14.1 (STATA Corp., College Station, Texas).

## Data Availability

The datasets generated and analysed during the current study are available from the corresponding author on reasonable request.
